# Value of dual-layer spectral detector computed tomography in the diagnosis of benign/malignant solid solitary pulmonary nodules and establishment of a prediction model

**DOI:** 10.3389/fonc.2023.1147479

**Published:** 2023-05-05

**Authors:** Xiaodong Xie, Kaifang Liu, Kai Luo, Youtao Xu, Lei Zhang, Meiqin Wang, Wenrong Shen, Zhengyang Zhou

**Affiliations:** ^1^ Department of Radiology, Nanjing Medical University Affiliated Cancer Hospital, Jiangsu Cancer Hospital, Jiangsu Institute of Cancer Research, Nanjing, China; ^2^ Department of Radiology, Nanjing Drum Tower Hospital, Clinical College of Nanjing Medical University, Nanjing, China; ^3^ Department of Thoracic Surgery, Jiangsu Cancer Hospital, Jiangsu Institute of Cancer Research, Nanjing Medical University Affiliated Cancer Hospital, Nanjing, China

**Keywords:** dual-layer spectral detector computed tomography, solitary pulmonary nodule, quantitative parameters, differential diagnosis, prediction model

## Abstract

**Objective:**

This study aimed to investigate the role of spectral detector computed tomography (SDCT) quantitative parameters and their derived quantitative parameters combined with lesion morphological information in the differential diagnosis of solid SPNs.

**Methods:**

This retrospective study included basic clinical data and SDCT images of 132 patients with pathologically confirmed SPNs (102 and 30 patients in the malignant and benign groups, respectively). The morphological signs of SPNs were evaluated and the region of interest (ROI) was delineated from the lesion to extract and calculate the relevant SDCT quantitative parameters, and standardise the process. Differences in qualitative and quantitative parameters between the groups were statistically analysed. A receiver operating characteristic (ROC) curve was constructed to evaluate the efficacy of the corresponding parameters in the diagnosis of benign and malignant SPNs. Statistically significant clinical data, CT signs and SDCT quantitative parameters were analysed using multivariate logistic regression to determine the independent risk factors for predicting benign and malignant SPNs, and the best multi-parameter regression model was established. Inter-observer repeatability was assessed using the intraclass correlation coefficient (ICC) and Bland–Altman plots.

**Results:**

Malignant SPNs differed from benign SPNs in terms of size, lesion morphology, short spicule sign, and vascular enrichment sign (*P<* 0.05). The SDCT quantitative parameters and their derived quantitative parameters of malignant SPNs (SAR_40keV_, SAR_70keV_, Δ_40keV_, Δ_70keV_, CER_40keV_, CER_70keV_, NEF_40keV_, NEF_70keV_, λ, NIC, NZ_eff_) were significantly higher than those of benign SPNs (*P<* 0.05). In the subgroup analysis, most parameters could distinguish between benign and adenocarcinoma groups (SAR_40keV_, SAR_70keV_, Δ_40keV_, Δ_70keV_, CER_40keV_, CER_70keV_, NEF_40keV_, NEF_70keV_, λ, NIC, and NZ_eff_), and between benign and squamous cell carcinoma groups (SAR_40keV_, SAR70_keV_, Δ_40keV_, Δ_70keV_, NEF_40keV_, NEF_70keV_, λ, and NIC). However, there were no significant differences between the parameters in the adenocarcinoma and squamous cell carcinoma groups. ROC curve analysis indicated that NIC, NEF_70keV_, and NEF_40keV_ had higher diagnostic efficacy for differentiating benign and malignant SPNs (area under the curve [AUC]:0.869, 0.854, and 0.853, respectively), and NIC was the highest. Multivariate logistic regression analysis showed that size (OR=1.138, 95% CI 1.022-1.267, *P*=0.019), Δ_70keV_ (OR=1.060, 95% CI 1.002-1.122, *P*=0.043), and NIC (OR=7.758, 95% CI 1.966-30.612, *P*=0.003) were independent risk factors for the prediction of benign and malignant SPNs. ROC curve analysis showed that the AUC of size, Δ_70keV_, NIC, and a combination of the three for differential diagnosis of benign and malignant SPNs were 0.636, 0.846, 0.869, and 0.903, respectively. The AUC for the combined parameters was the largest, and the sensitivity, specificity, and accuracy were 88.2%, 83.3% and 86.4%, respectively. The SDCT quantitative parameters and their derived quantitative parameters in this study exhibited satisfactory inter-observer repeatability (ICC: 0.811-0.997).

**Conclusion:**

SDCT quantitative parameters and their derivatives can be helpful in the differential diagnosis of benign and malignant solid SPNs. The quantitative parameter, NIC, is superior to the other relevant quantitative parameters and when NIC is combined with lesion size and Δ_70keV_ value for comprehensive diagnosis, the efficacy could be further improved.

## 1 Introduction

In 2020, there were approximately 2.2 million new cases of lung cancer worldwide, accounting for 11.4% of all malignant tumours, and approximately 1.8 million deaths, accounting for 18.0% of all deaths due to malignant tumours (1, 2). The 5-year survival rate of patients with stage I lung cancer is 60%-80%, while that of stage IV patients is only 5.3%. Therefore, early detection, diagnosis, and treatment are important for patients with lung cancer (3). The common manifestations in the early stages of lung cancer, solitary pulmonary nodules (SPNs) are round or round-like pulmonary parenchymal lesions with relatively clear boundaries and a diameter of ≤ 30 mm (4). The detection of SPNs is increasing owing to the popularisation of CT examinations (5). However, due to the atypical clinical symptoms and incidental nature in some patients, SPNs can manifest as primary lung cancer, pulmonary metastatic nodules, infection foci, or other benign lesions. In addition, the malignancy rate of SPNs is only 30%-40% (6). Therefore, quick and accurate diagnosis of malignant SPNs and the provision of standardised diagnosis and treatment guidance are challenging.

Detection of SPNs by low-dose spiral computed tomography (LDCT) screening and early treatment interventions decreases the mortality of lung cancer patients by 20% (7); however, LDCT plays little role in the qualitative diagnosis of SPNs (8). Conventional CT plays an important role in evaluating the radiological signs and enhancement characteristics of SPNs, but some benign and malignant SPNs have highly similar lesion morphologies and enhancement patterns, and thus pose a great challenge in differentiating benign and malignant SPNs (9). ^18^F-fluorodeoxyglucose positron emission tomography/computed tomography (^18^F-FDG PET/CT) can reflect the internal metabolism of SPNs based on conventional CT, but false positive results due to inflammation, infection and other factors are difficult to avoid (10). In addition, the high radiation dose and the expensiveness of ^18^F-FDG PET/CT limits its application and promotion in China. Needle biopsy has high accuracy in differentiating benign from malignant SPNs. However, as an invasive examination, patients not only bear the risk of complications (pneumothorax, bleeding, infection, needle dissemination, and implantation) but also economic and mental stress. Therefore, it is very important and urgent to explore a non-invasive and accurate method for differentiating benign from malignant SPNs.

Dual-layer spectral detector computed tomography (SDCT), a milestone in the development of CT, uses materials to produce different energy information at various X-ray energies to convert simple CT images to functional imaging. In contrast to conventional CT, SDCT can also obtain quantitative parameters such as iodine concentration (IC), the slope of the spectral attenuation curve (λ), Z-effective (Z_eff_), and electron density (ED), in addition to CT values. Currently, SDCT is gradually being applied and promoted in many clinical fields (11, 12). In clinical oncology, SDCT is recognised for its ability to differentiate benign and malignant primary lesions, malignant tumour subtypes, primary lesions from metastases, and lymph node metastasis(LNM) predictions (13–15). Nonetheless, studies focusing on the differential diagnoses of benign and malignant pulmonary nodules are limited. Published literature focused on the quantitative parameters of SDCT in the differential diagnosis of benign and malignant SPNs (16–18). However, the clinical data of the patients and the morphological characteristics of the tumour were not included in the comprehensive analysis. Hence, this study aimed to 1) explore the importance of SDCT quantitative parameters and their derivatives, combined with morphological information of the lesion and clinical data of patients in the differential diagnosis of benign and malignant solid SPNs and 2) establish a prediction model for malignant SPNs to assist in the development of its best treatment strategy.

## 2 Materials and methods

### 2.1 Study population

The study was approved by the institutional ethics committee of Jiangsu Cancer Hospital (NO.ZN202212, Nanjing, Jiangsu, China) and the requirement for individual consent for this retrospective analysis was waived.

**Figure 1:**
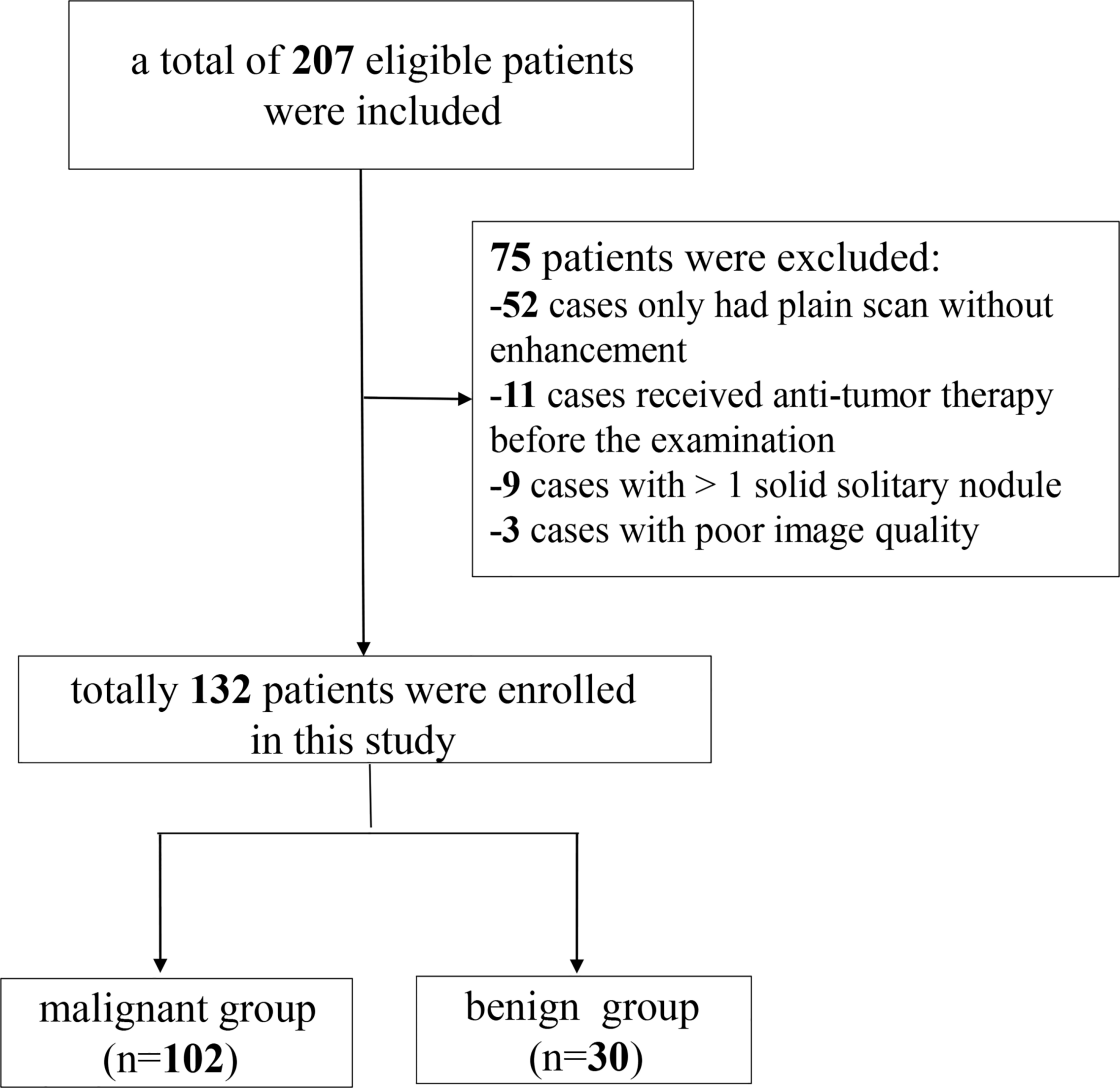
Patients enrolment flowchart.

Data from 207 patients with newly diagnosed SPNs who underwent spectral CT examination in our hospital between September 2021 and August 2022 were retrospectively collected. The inclusion criteria for the participants were as follows: (i) SDCT chest scan performed in our hospital; (ii) patients diagnosed with solid SPNs with a lesion diameter of > 8 mm and ≤ 30 mm; and (iii) definite pathological results confirmed by surgery or puncture. The exclusion criteria were: (i) plain chest scan without enhanced scan (n = 52); (ii) anti-tumour therapy before SDCT examination (n = 11); (iii) patients with more than one solid solitary nodules (n = 9); and (iv) poor image quality (n = 3). Finally, 132 patients were enrolled in this study, including 102 with malignant SPNs and 30 with benign SPNs. A flow chart of patient inclusion and exclusion is shown in **Figure 1**.

Based on tumour pathology, the study participants were divided into two groups: malignant (n = 102) and benign (n = 30). The histopathological subtypes of malignant SPNs of the patients included are adenocarcinoma (n = 76); squamous cell carcinoma (n = 21); small cell lung cancer (n = 4); adenosquamous carcinoma (n = 1). Among 30 patients with benign SPNs, inflammation (n = 9), pulmonary hamartoma (n = 8), pulmonary tuberculosis (n = 8), inflammatory pseudotumour (n = 2), alveolar cytoma (n = 1), congenital cystic adenomatoid malformation (n = 1), and collagen nodules with lymphoproliferation (n = 1) were observed.

### 2.2 SDCT image acquisition

All patients underwent the same routine SDCT protocol (IQon; Philips Healthcare, Best, Netherlands). The patients were placed in the supine position, and the scanning range was from the thoracic entrance to the level of the costophrenic angle. The basic scanning parameters were: tube voltage, 120 kVp; tube current modulation; 3D modulation; collimator width, 64×0.625 mm; matrix, 512×512; and scanning field of view, 372 mm; pitch, 0.90; rotation time, 0.50 s. The slice thickness was 5 mm for scanning and 1 mm for reconstruction. Contrast medium (ioversol, 3.0 ml/kg; iodine, 350 mg/ml; HengRui Medicine, Jiangsu, China) was injected intravenously in the anterior elbow at a flow rate of 2.5-3.0 ml/s, followed by 20 ml normal saline at the same flow rate. A contrast-enhanced chest scan was performed 50 s later after the injection.

### 2.3 Radiological analysis of SPNs on SDCT

#### 2.3.1 Analysis of the morphological characteristics

The images of all SPNs cases were interpreted and analysed by two radiologists (with 4 years and 8 years of work experience) blinded to the pathological findings. For analysing the morphological features of SPNs, different window widths, window levels, thin-slice scans, and multi-planar reconstruction techniques were used to observe the lesion location, shape, lobulation, spiculation, pleural indentation, vascular convergence sign, air bronchogram, vacuole sign, calcification, and other imaging signs. When perceptions were inconsistent, a consensus was reached through consultation.

#### 2.3.2 Acquisition and analysis of SDCT quantitative parameters

All images were imported into a Philips workstation (IntelliSpace Portal, Philips Healthcare), and the analysis and processing of the images were performed using the software in the workstation (Spectral CT Viewer, Philips Healthcare). Image analysis was performed by a radiologist (with 4 years of radiology experience) and supervised by a senior radiologist (with 8 years of radiology experience). All SPNs image data were acquired within the mediastinal window. Regions of interest (ROI) were manually delineated at three consecutive levels, including the largest level of the SPNs and its adjacent upper and lower levels. Areas such as calcification, vessels, cavities, atelectasis, and necrosis, which may affect measurements, were avoided. An average of three measurements for each case was taken as the final data point for the analysis. The ROI of the aorta at similar levels was obtained using the same method and used for the normalisation of SDCT quantitative parameters. Subsequently, a series of parameters was obtained.

The parameters obtained in our study were: CT values of SPNs at virtual non-contrast (VNC) and enhancement (40keV and 70keV) were recorded as CT_SPN-VNC_, CT_SPN-40keV_ and CT_SPN-70keV_, respectively. The CT values of the aorta at the VNC (CT_aorta-VNC_) and enhancement at 70keV (CT_aorta_) were recorded and used as a reference. The following formulas were used to calculate the ratio of lesion to aortic virtual plain scan (SAR_VNC_), the ratio of the lesion to aortic contrast enhancement (SAR_40keV_, SAR_70keV_), the difference in CT value between lesion enhanced and virtual plain scan (Δ_40keV_, Δ_70keV_), and the contrast enhancement ratio (CER_40keV_, CER_70keV_). Standardised enhancement value scores (NEF_40keV_, NEF_70keV_) and spectral curve slopes (λ) (19).


$${\text{SAR}}_{\text{VNC}}={\text{CT}}_{\text{SPN}-\text{VNC}}/{\text{CT}}_{\text{aorta}-\text{VNC}}$$

$${\text{SAR}}_{40\text{keV}/70\text{keV}}={\text{CT}}_{\text{SPN}-40\text{keV}/70\text{keV}}/{\text{CT}}_{\text{aorta}}$$

$$\Delta_{40\text{keV}/70\text{keV}}={\text{CT}}_{\text{SPN}-40\text{keV}/70\text{keV}}-{\text{CT}}_{\text{SPN}-\text{VNC}}$$

$${\text{CER}}_{40\text{keV}/70\text{keV}}=\Delta_{40\text{keV}/70\text{keV}}/{\text{CT}}_{\text{SPN}-\text{VNC}}$$

$${\text{NEF}}_{40\text{keV}/70\text{keV}}=\Delta_{40\text{keV}/70\text{keV}}/({\text{CT}}_{\text{aorta}}-{\text{CT}}_{\text{aorta}-\text{VNC}})$$

$$\lambda =({\text{CT}}_{\text{SPN}-40\text{keV}}-{\text{CT}}_{\text{SPN}-70\text{keV}})/(70-40)$$

To account for the haemodynamic variation between patients, the IC (mg/mL), Z_eff_ and ED values were normalised to the aorta. The normalised iodine concentration (NIC), normalised electron density (NED), and normalised effective atomic number (NZ_eff_) were calculated according to the following formula:


$$\text{NIC}={\text{IC}}_{\text{SPN}}/{\text{IC}}_{\text{aorta}}$$

$$\text{NED}={\text{NED}}_{\text{SPN}}/{\text{NED}}_{\text{aorta}}$$

$${\text{NZ}}_{\text{eff}}={\text{Z}}_{\text{ef}f-\text{SPN}}/{\text{Z}}_{\text{eff}-\text{aorta}}$$

To assess inter-observer reproducibility and variability, 50% of the study participants (66/132) were randomly selected, and the previous measurement procedure was repeated by another radiologist. Interobserver agreement was assessed using Bland–Altman plots and intraclass correlation coefficients (ICC).

### 2.4 Statistical analyses

The data were statistically analysed using SPSS 22 (SPSS, Inc., Chicago, IL, USA) and MedCalc15 (MedCalc Software, Mariakerke, Belgium). Continuous variables were presented as mean ± standard deviation. The basic clinical data and SDCT morphological characteristics were analysed using the chi-square test or Fisher’s exact test. The SPNs SDCT quantitative parameters and derived parameters were tested for normality using the Kolmogorov–Smirnov method, followed by the Levene test for homogeneity of variance. An independent sample t-test or Mann–Whitney U test was used to compare multiparameter differences between benign and malignant SPNs. One-way Analysis of Variance (ANOVA) was used to compare the pairwise differences between the benign and adenocarcinoma groups, benign and squamous cell carcinoma groups, and adenocarcinoma and squamous cell carcinoma groups. Receiver operating characteristic (ROC) curve analysis was used to determine the area under the curve (AUC), accuracy, sensitivity, and specificity of the different parameters in predicting benign and malignant SPNs. The clinical data of SPNs, SDCT morphological features, SDCT quantitative parameters, and their derived parameters with statistical differences were analysed by multivariate logistic regression to determine the independent risk factors for predicting benign and malignant SPNs, and the best multi-parameter regression prediction model was established. The inter-observer agreement of spectral CT parameters was evaluated using ICC (0.000-0.200: poor; 0.201-0.400: general; 0.401-0.600: medium; 0.601-0.800: good; 0.801-1.000: excellent) and the Bland–Altman plot evaluation.

## 3 Results

### 3.1 Basic clinical data of the study participants

Of the 132 pathologically confirmed solid SPNs patients included, 102 showed malignancy (42 males; 59 females) with an average age of 64.147 ± 9.491 years, and 61 of them were smokers. The 30 patients with benign tumours (14 males; 16 females) had an average age of 60.233 ± 10.047 years, and 12 of them were smokers. There were no significant differences in age (*P* = 0.052), sex (*P* = 0.279), or smoking history (*P* = 0.985) between the benign and malignant groups **Table 1**.

**Table 1:** Basic clinical data of the patients.

	Benign group (n=30)	Malignant group (n=102)	*P*-value
Age (years)	60.233 ± 10.047	64.147 ± 9.491	0.052
Sex			0.279
male	14	43	
female	16	59	
Smoking			0.985
yes	12	61	
no	18	41	

### 3.2 Morphological features of benign and malignant SPNs

**Table 2** shows the differences in the morphological features of benign and malignant SPNs identified using SDCT. The radiological findings of the two groups were analysed based on the following 10 aspects: location, size, lesion shape, lobulation, short spiculation, pleural indentation, vascular convergence sign, air bronchogram, vacuole sign, and calcification. There were significant differences in lesion size (*P* = 0.04), shape (*P* = 0.014), short spiculation sign (*P* = 0.034), and vascular convergence sign (*P* = 0.015) between the benign and malignant groups. The difference in the location, lobulation sign, pleural indentation sign, air bronchogram sign, vacuole sign, and calcification were not significant between the two groups (*P* > 0.05).

**Table 2:** Radiological morphological signs of benign and malignant SPNs on SDCT images.

	Benign group (n=30)	Malignant group (n=102)	*P-*value
**Location (lung)**			0.496
upper lobe of left	9	24	
lower lobe of left	6	14	
upper lobe of right	6	38	
middle lobe of right	3	7	
lower lobe of right	6	19	
**size**	15.772 ± 5.661 mm	18.225 ± 5.689 mm	0.040*
**Lesion shape**			0.014*
round	10	14	
irregular	20	88	
**Lobulation**			0.123
yes	17	73	
no	13	29	
**Short spiculation**			0.034*
yes	9	53	
no	21	49	
**Pleural indentation**			0.095
yes	6	37	
no	24	65	
**Vascular convergence sign**			0.015*
yes	8	53	
no	22	49	
**Air bronchogram sign**			0.053
yes	5	36	
no	25	66	
**Vacuole sign**			0.296
yes	1	12	
no	29	90	
**Calcification**			0.694
yes	3	7	
no	27	95	

*p value<0.05.

### 3.3 Comparison of SDCT quantitative parameters between the benign and malignant groups

**Figure 2:**
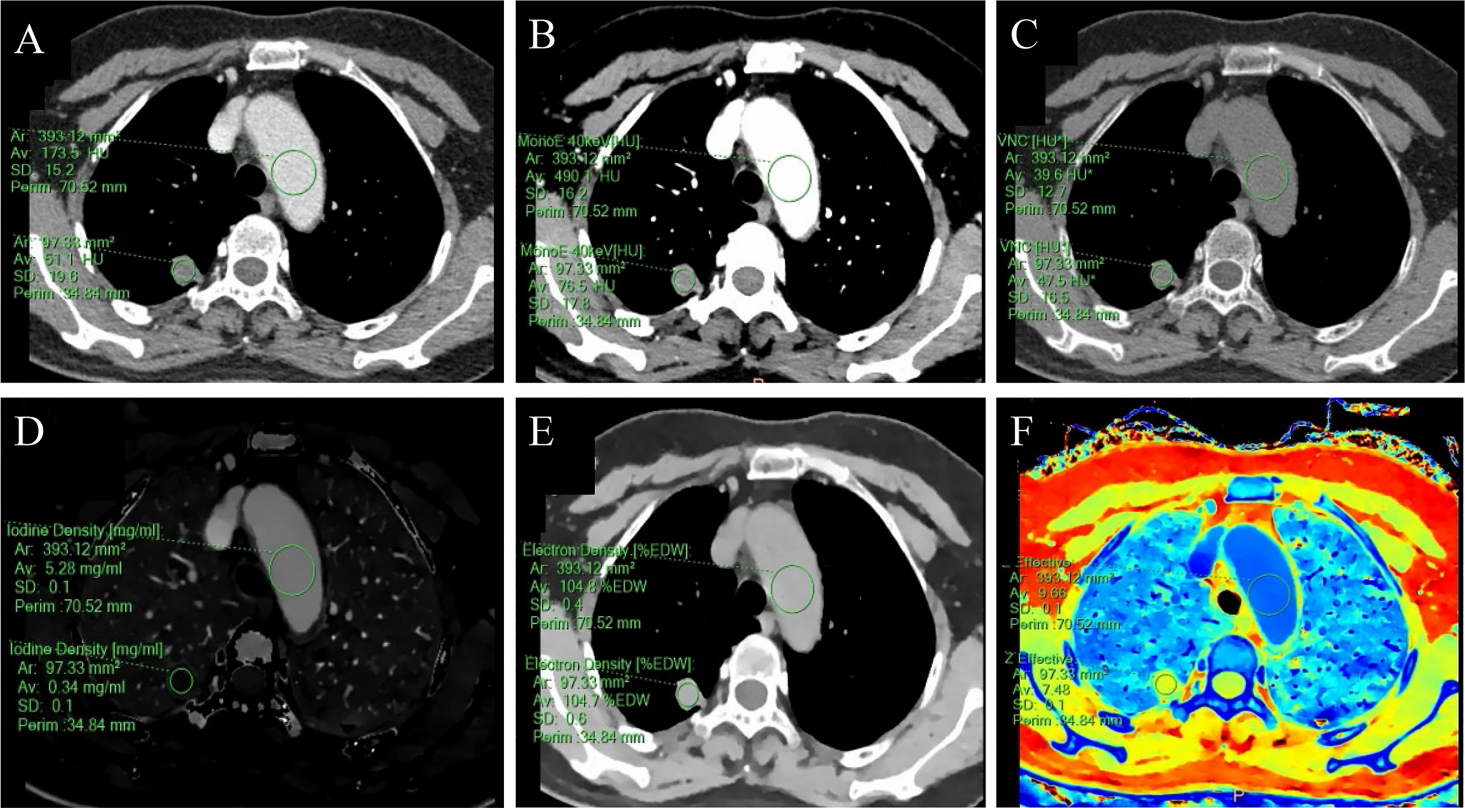
A 59-year-old female with a solid SPN in the posterior segment of the right upper lobe had pathologically confirmed hamartoma. The corresponding quantitative parameter maps of SDCT at 70keV, 40keV, virtual non-contrast (VNC), iodine density (IC), electron density (ED) and effective atomic number (Zeff) are shown in **(A-F)**, respectively. The region of interest (ROI) outlined by the green circle reflects the corresponding SDCT quantitative parameters of the lesion and the aorta at the same level. In this case: CT_SPN-70keV_ = 51.1 HU, CT_SPN-40keV_ = 76.5 HU, CT_SPN-VNC_ = 47.5 HU, IC_SPN_ = 0.34 mg/ml, ED_SPN_ = 104.7% EDW, Z_eff-spn_ = 7.48, CT _aorta-70keV_ = 173.5 HU, CT _aorta-40keV_ = 490.1 HU, CT _aorta-VNC_ = 39.6 HU, IC_aorta_ = 5.28 mg/ml, ED_aorta_ = 104.8% EDW, Z_eff-aorta_ = 9.66.

**Figure 3:**
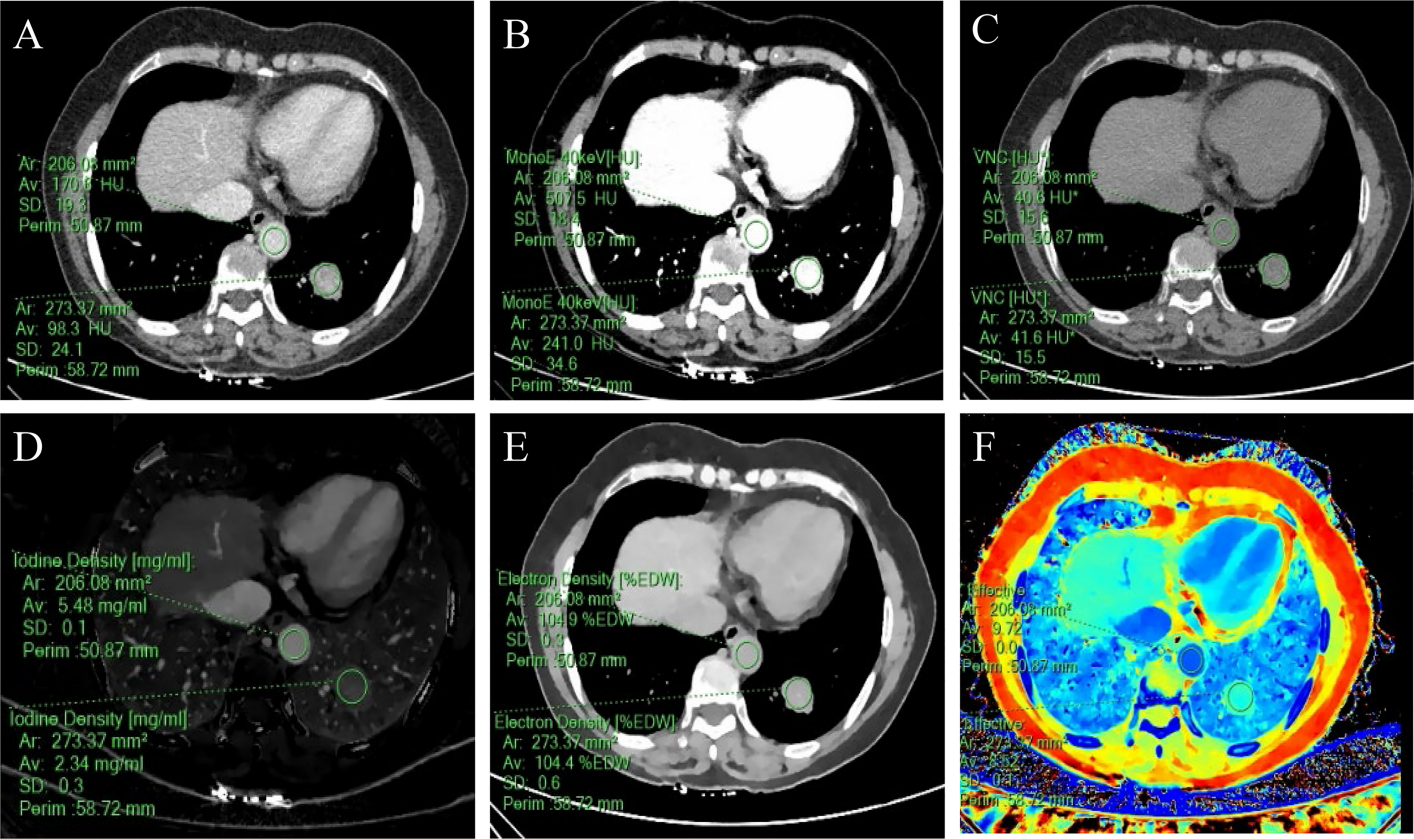
A 57-year-old woman with a solid SPN in the lateral basal segment of the left lower lobe had pathologically confirmed adenocarcinoma. The corresponding quantitative parameter maps of SDCT at 70keV, 40keV, virtual non-contrast (VNC), iodine density (IC), electron density (ED) and effective atomic number (Zeff) are shown in **(A-F)**, respectively. The region of interest (ROI) outlined by the green circle reflects the corresponding SDCT quantitative parameters of the lesion and the aorta at the same level. In this case: CT_SPN-70keV_ = 98.3 HU, CT_SPN-40keV_ = 241.0 HU, CT_SPN-VNC_ = 41.6 HU, IC_SPN_ = 2.34 mg/ml, ED_SPN_ = 104.4% EDW, Z_eff-spn_= 8.52, CT_aorta-70keV_ = 170.6 HU, CT_aorta-40keV_ = 507.5 HU, CT_aorta-VNC_ = 40.6 HU, IC_aorta_ = 5.48 mg/ml, ED_aorta_ = 104.9% EDW, Z_eff-aorta_ = 9.72.

The SDCT quantitative parameters were obtained by ROI delineation and then calculated. 13 quantitative parameter indices were included in this study for statistical analysis, and the specific quantitative parameter analysis findings are shown in **Table 3**. Except for SAR_VNC_ and NED (*P* = 0.114 and 0.208, respectively), all other parameters (SAR_40keV_, SAR_70keV_, Δ_40keV_, Δ_70keV_, CER_40keV_, CER_70keV_, NEF_40keV_, NEF_70keV_, λ, NIC, and NZ_eff_) were significantly higher in the malignant group than in the benign group (*P*< 0.05). A schematic diagram of ROI delineation and quantitative parameter acquisition for typical cases of benign and malignant SPNs are shown in **Figures 2** and **3**, respectively.

**Table 3:** Comparison of SDCT quantitative parameters between benign and malignant SPNs.

Parameters	Benign group (n=30)	Malignant group (n=102)	*P-*value
SAR_VNC_	0.707 ± 0.357	0.785 ± 0.229	0.114
SAR_40keV_	0.512 ± 0.198	0.841 ± 0.267	<0.001*
SAR_70keV_	0.240 ± 0.098	0.351 ± 0.102	<0.001*
Δ_40keV_	83.884 ± 43.997	146.955 ± 46.532	<0.001*
Δ_70keV_	21.944 ± 13.584	42.059 ± 14.872	<0.001*
CER_40keV_	3.262 ± 2.480	5.241 ± 3.577	<0.001*
CER_70keV_	0.830 ± 0.699	1.453 ± 1.035	<0.001*
NEF_40keV_	0.462 ± 0.212	0.872 ± 0.364	<0.001*
NEF_70keV_	0.120 ± 0.067	0.247 ± 0.110	<0.001*
λ	1.990 ± 1.091	3.489 ± 1.141	<0.001*
NIC	0.134 ± 0.064	0.260 ± 0.101	<0.001*
NED	0.961 ± 0.089	0.981 ± 0.011	0.208
NZ_eff_	0.764 ± 0.040	0.804 ± 0.080	<0.001*

*p value<0.05.

### 3.4 Differences in SDCT quantitative parameters among benign, adenocarcinoma, and squamous cell carcinoma groups

The SDCT quantitative parameters among the benign, adenocarcinoma, and squamous cell carcinoma groups are shown in **Table 4**. Except for SAR_VNC_ and NED (*P* = 0.788 and 0.572, respectively), the other parameters (SAR_40keV_, SAR_70keV_, Δ_40keV_, Δ_70keV_, CER_40keV_, CER_70keV_, NEF_40keV_, NEF_70keV_, λ, NIC, and NZ_eff_) were significantly higher in the adenocarcinoma group than in the benign group (*P*< 0.05). Similarly, except for SAR_VNC_, CER_40keV_, CER_70keV_, NED, and NZ_eff_ (*P* = 0.209, 0.434, 0.230, 0.404, and 0.237), the other eight parameters (SAR_40keV_, SAR_70keV_, Δ_40keV_, Δ_70keV_, NEF_40keV_, NEF_70keV_, λ, and NIC) were significantly higher in the squamous cell carcinoma group than in the benign group (*P*< 0.05). There were no significant differences in the SDCT quantitative parameters between the adenocarcinoma and squamous cell carcinoma groups (*P* > 0.05).

**Table 4:** Differences in SDCT quantitative parameters among the sub-groups.

Parameters	Benign group (n=30)	Adenocarcinoma group (n=76)	Squamous cell carcinoma group(n=21)		*P-*value	
				Benign vs Adenocarcinoma	Benign Vs Squamous cell carcinoma	Adenocarcinoma vs Squamous cell carcinoma
SAR_VNC_	0.707 ± 0.357	0.765 ± 0.242	0.844 ± 0.177	0.788	0.209	0.282
SAR_40keV_	0.512 ± 0.198	0.852 ± 0.288	0.800 ± 0.207	<0.001*	<0.001*	0.739
SAR_70keV_	0.240 ± 0.098	0.352 ± 0.110	0.344 ± 0.086	<0.001*	<0.001*	0.754
Δ_40keV_	83.884 ± 43.997	150.456 ± 46.649	141.389 ± 47.793	<0.001*	<0.001*	0.824
Δ_70keV_	21.944 ± 13.548	42.541 ± 13.827	39.600 ± 15.328	<0.001*	<0.001*	0.812
CER_40keV_	3.262 ± 2.480	5.374 ± 3.703	4.076 ± 1.744	0.003*	0.434	0.075
CER_70keV_	0.830 ± 0.699	1.528 ± 1.090	1.138 ± 0.545	0.001*	0.230	0.078
NEF_40keV_	0.462 ± 0.212	0.894 ± 0.398	0.795 ± 0.249	<0.001*	0.001*	0.244
NEF_70keV_	0.120 ± 0.067	0.254 ± 0.120	0.221 ± 0.073	<0.001*	0.001*	0.197
λ	1.990 ± 1.091	3.529 ± 1.101	3.320 ± 1.109	<0.001*	<0.001*	0.442
NIC	0.134 ± 0.064	0.264 ± 0.108	0.229 ± 0.075	<0.001*	<0.001*	0.264
NED	0.961 ± 0.089	0.980 ± 0.112	0.985 ± 0.009	0.572	0.404	0.191
NZ_eff_	0.764 ± 0.040	0.809 ± 0.088	0.789 ± 0.528	0.005*	0.237	0.271

*p value<0.05.

### 3.5 Diagnostic performance of SDCT quantitative parameters

**Figure 4:**
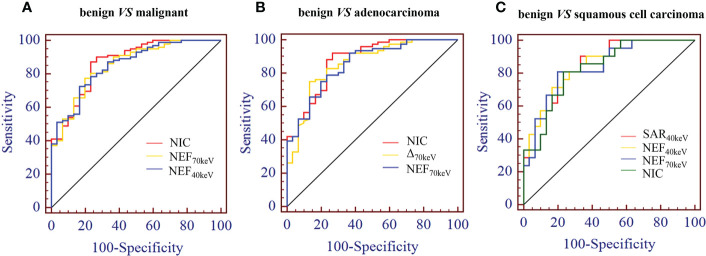
**(A)** Receiver operating characteristic curve (ROC) showed that NIC, NEF_70keV_ and NEF_40keV_ had the highest diagnostic efficiency in benign and malignant SPNS, and the AUC were 0.869, 0.854 and 0.853, respectively, of which NIC was the highest. **(B)** The three parameters with the highest diagnostic efficiency were NIC, Δ_70keV_ and NEF_70keV_, and their AUC were 0.876, 0.857 and 0.854, respectively. NIC still had the highest detection efficiency. **(C)** SAR_40keV_, NEF_40keV_ and NEF_70keV_ were the three parameters with the highest diagnostic efficiency to distinguish benign SPN from the squamous cell carcinoma group. The AUC were 0.849, 0.849 and 0.837, respectively. The diagnostic efficiency of NIC was 0.832, which was second only to the above three parameters.

The diagnostic performance of SDCT quantitative parameters for different groups is shown in **Tables 5**–**7** and **Figure 4**. Among the multiple quantitative parameters, NIC, NEF_70keV_ and NEF_40keV_ had the highest diagnostic efficiency in differentiating benign from malignant SPNs (AUC = 0.869, 0.854, and 0.853, respectively), and the AUC of NIC was the highest. When the cut-off value of NIC was 0.165, the sensitivity was 87.3%, the specificity was 76.7%, the positive prediction rate was 92.7%, the negative prediction rate was 63.9%, and the accuracy was 84.2%. NIC, Δ_70keV_ and NEF_70keV_ had the highest diagnostic efficiency in distinguishing benign SPNs from adenocarcinoma (AUC = 0.876, 0.857, and 0.854, respectively) and the NIC exhibited the highest detection efficiency. When the cut-off value of the NIC was 0.158, the sensitivity, specificity, positive predictive value, negative predictive value, and accuracy were 92.1%, 73.3%, 89.7%, 78.6%, and 86.0%, respectively. The three parameters with the highest diagnostic efficiency to distinguish between benign SPNs and squamous cell carcinoma were SAR_40kev_, NEF_40keV_ and NEF_70keV_ (AUC = 0.849, 0.849, and 0.837, respectively). The diagnostic efficiency of the NIC was 0.832, which was second only to the above three parameters. In general, NIC is the best quantitative index for differentiating benign from malignant SPNs and for the comparison between the different subgroups.

**Table 5:** Diagnostic efficacy of SDCT quantitative parameters in benign and malignant groups.

Parameters	AUC	Cut-off value	Sensitivity (%)	Specificity (%)	Positive predictive value (%)	Negative predictive value (%)	Accuracy (%)	*P*-value
SAR_40keV_	0.848	0.617	79.4	76.7	92.0	52.3	78.2	<0.001*
SAR_70keV_	0.783	0.331	53.9	90.0	94.8	36.5	61.7	<0.001*
Δ_40keV_	0.836	113.217	75.5	80.0	92.8	49.0	75.9	<0.001*
Δ_70keV_	0.846	33.000	71.6	86.7	94.8	47.3	74.4	<0.001*
CER_40keV_	0.737	3.7354	57.8	80.0	89.4	34.8	61.7	<0.001*
CER_70keV_	0.750	1.105	53.9	83.3	91.7	34.7	60.2	<0.001*
NEF_40keV_	0.853	0.650	72.5	83.3	92.5	46.2	73.7	<0.001*
NEF_70keV_	0.854	0.168	77.5	80.0	91.9	50.0	76.7	<0.001*
λ	0.831	2.662	76.5	80.0	92.9	50.0	76.7	<0.001*
NIC	0.869	0.165	87.3	76.7	92.7	63.9	84.2	<0.001*
NZ_eff_	0.695	0.814	36.3	96.7	94.8	36.5	61.7	<0.001*.

*p value<0.05.

**Table 6:** Diagnostic efficacy of SDCT quantitative parameters in benign and adenocarcinoma groups.

Parameters	AUC	Cut-off value	Sensitivity (%)	Specificity (%)	Positive predictive value (%)	Negative predictive value (%)	Accuracy (%)	*P*-value
SAR_40keV_	0.842	0.656	75.0	80.0	89.1	54.8	74.8	<0.001*
SAR_70keV_	0.771	0.331	50.0	90.0	92.7	41.5	60.7	<0.001*
Δ_40keV_	0.849	113.217	78.9	80.0	90.9	60.0	78.5	<0.001*
Δ_70keV_	0.857	33.0	75.0	86.7	93.4	57.8	77.6	<0.001*
CER_40keV_	0.747	3.7354	61.8	80.0	87.0	44.2	65.4	<0.001*
CER_70keV_	0.770	1.050	61.8	80.0	88.7	45.3	66.4	<0.001*
NEF_40keV_	0.850	0.650	72.4	83.3	91.5	53.2	73.8	<0.001*
NEF_70keV_	0.854	0.155	78.9	76.7	89.6	59.0	77.6	<0.001*
λ	0.842	2.662	78.9	80.0	90.9	60.0	78.5	<0.001*
NIC	0.876	0.158	92.1	73.3	89.7	78.6	86.0	<0.001*
NZ_eff_	0.705	0.814	39.5	96.7	96.9	39.2	56.1	<0.001*

*p value<0.05.

**Table 7:** Diagnostic efficacy of SDCT quantitative parameters in benign and squamous cell carcinoma groups.

Parameters	AUC	Cut-off value	Sensitivity (%)	Specificity (%)	Positive predictive value (%)	Negative predictive value (%)	Accuracy (%)	*P*-value
SAR_40keV_	0.849	0.617	81.0	76.7	68.0	84.6	75.0	<0.001*
SAR_70keV_	0.792	0.326	66.7	86.7	77.8	78.8	76.9	<0.001*
Δ_40keV_	0.811	113.217	76.2	80.0	72.7	82.8	76.9	<0.001*
Δ_70keV_	0.817	31.93	71.4	83.3	71.4	80.0	75.0	<0.001*
NEF_40keV_	0.849	0.650	71.4	83.3	75.0	80.6	76.9	<0.001*
NEF_70keV_	0.837	0.168	81.0	80.0	70.8	85.2	76.9	<0.001*
λ	0.806	2.662	76.2	80.0	72.7	82.8	76.9	<0.001*
NIC	0.832	0.165	81.0	76.7	70.8	85.2	76.9	<0.001*

*p value<0.05.

### 3.6 Morphological features in combination with SDCT quantitative parameters for the differential diagnosis of benign and malignant SPNs

**Figure 5:**
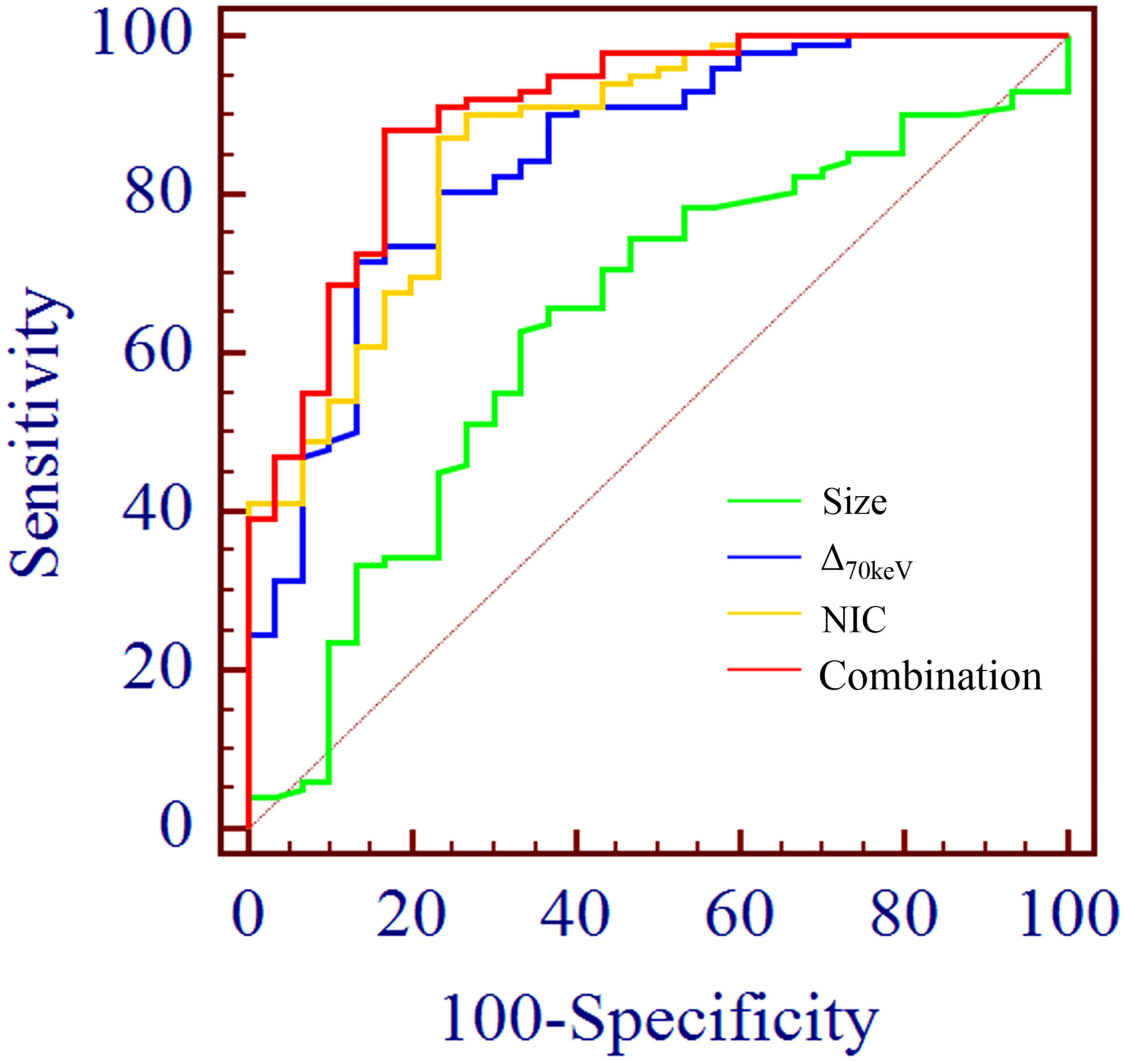
ROC curve analysis showed the AUC of size, Δ_70keV_, NIC and combination of the three for differential diagnosis of benign and malignant SPN were 0.636, 0.846, 0.869 and 0.903, respectively. The AUC (0.903) of the combination parameters (size +Δ_70keV_+NIC) was the largest, which was superior to NIC (AUC = 0.869), the highest diagnostic efficiency among independent risk factors.

Binary logistic regression analysis performed with the SPNs size, shape, short spiculation, vascular convergence, and 11 quantitative parameters (SAR_40keV_, SAR_70keV_, Δ_40keV_, Δ_70keV_, CER_40keV_, CER_70keV_, NEF_40keV_, NEF_70keV_, λ, NIC, and NZ_eff)_ that were different between benign and malignant groups as independent variables, and benign or malignant SPNs as the dependent variables showed that lesion size (OR = 1.138, 95% CI 1.022-1.267, *P*=0.019), Δ_70keV_ (OR=1.060, 95% CI 1.002-1.122, *P*=0.043), and NIC (OR=7.758, 95% CI 1.966-30.612, *P*=0.003) were independent risk factors for the prediction of benign and malignant SPNs. ROC curve analysis showed that the AUC of size, Δ_70keV_, NIC, and a combination of the three for differential diagnosis of benign and malignant SPNs were 0.636, 0.846, 0.869, and 0.903, respectively. The AUC of the combination of the parameters (size +Δ_70keV_+NIC) was the largest (0.903) when the cut-off value was 0.728, the sensitivity, specificity, positive predictive rate, negative predictive rate and accuracy were 88.2%, 83.3%, 93.8%, 66.7% and 86.4%, respectively. The probability prediction model was logit(*P*) = -6.707 + 0.129size+0.059Δ_70keV_+2.049NIC. The details are presented in **Table 8** and **Figure 5**.

**Table 8:** Diagnostic efficacy of independent risk factors and the combination model.

Parameters	AUC	Cut-off value	Sensitivity (%)	Specificity (%)	Positive predictive value (%)	Negative predictive value (%)	Accuracy (%)	*P*-value
size	0.636	16.250	62.7	66.7	86.7	35.1	63.9	0.021*
Δ_70keV_	0.846	33.000	71.6	86.7	94.8	47.3	74.4	<0.001*
NIC	0.869	0.165	87.3	76.7	92.7	63.9	84.2	<0.001*
combination model	0.903	0.728	88.2	83.3	93.8	66.7	86.4	<0.001*

*p value<0.05.

### 3.7 Assessment of inter-observer agreement

**Figure 6:**
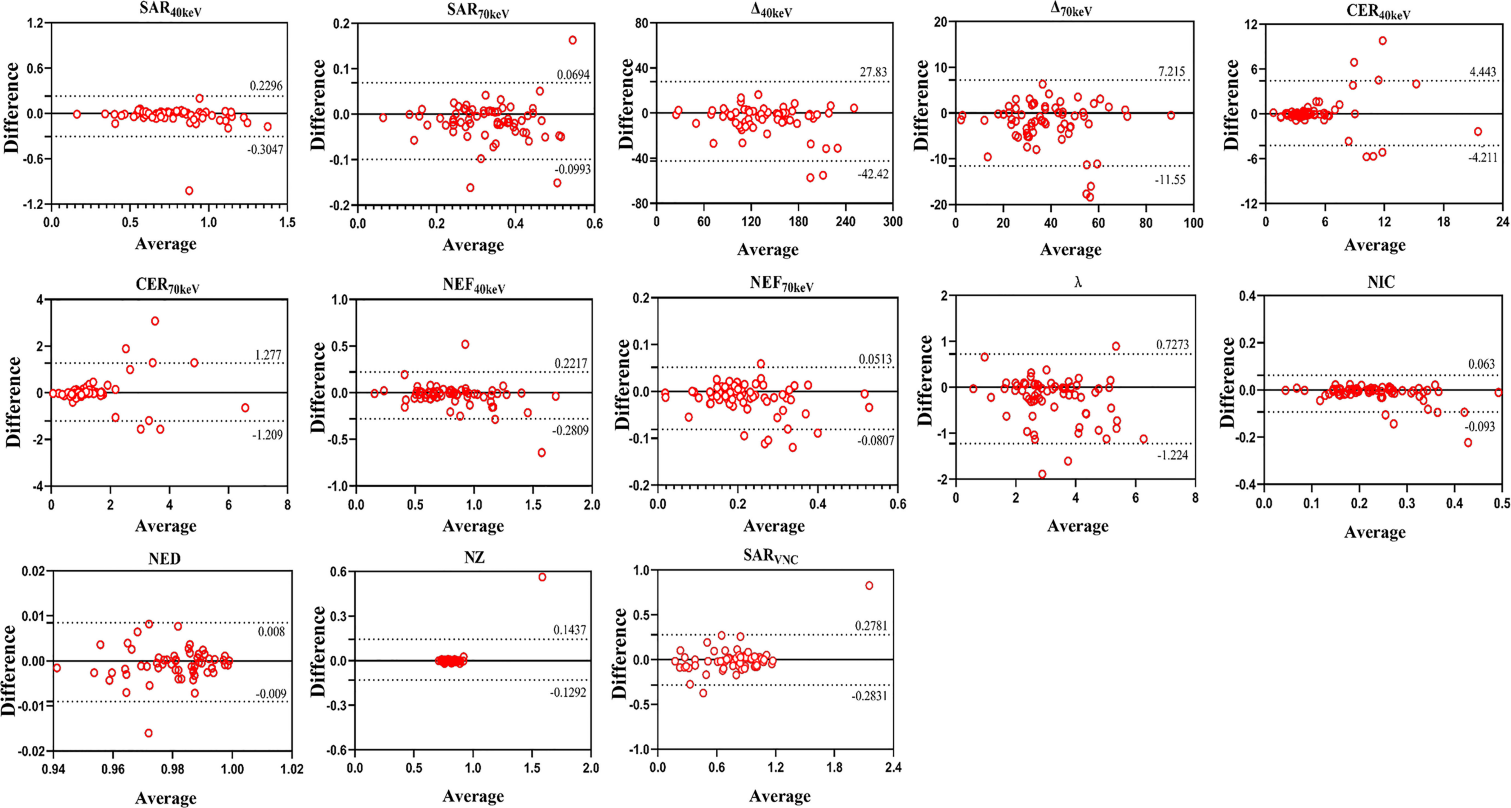
Bland–Altman plots of inter-observer differences in SDCT-related parameters.

All SDCT parameters showed good inter-observer agreement for assessing SPNs, with ICC values between 0.811-0.997 (**Table 9**, **Figure 6**).

**Table 9:** Repeatability of multi-parameters between observers.

Parameters	ICC (95% CI)	Parameters	ICC (95% CI)
SAR_40keV_	0.849 (0.764-0.905)	SAR_70keV_	0.910(0.858-0.944)
Δ_40keV_	0.939(0.902-0.962)	Δ_70keV_	0.957(0.931-0.974)
CER_40keV_	0.820(0.722-0.886)	CER_70keV_	0.845(0.759-0.902)
NEF_40keV_	0.915 (0.866-0.947)	NEF_70keV_	0.941 (0.906-0.964)
λ	0.916(0.867-0.948)	NIC	0.900(0.842-0.938)
NED	0.997(0.996-0.988)	NZ_eff_	0.811(0.708-0.880)
SAR_VNC_	0.898(0.839-0.936)		

## 4 Discussion

A multi-centre retrospective study by Tanner et al. reported that up to 35% of patients with benign pulmonary nodules received surgical treatment, which not only increased the risk of complications and medical costs but also unnecessary psychological stress and social burden (20). Interestingly, our study suggests that SDCT quantitative parameters combined with morphological features are helpful in the diagnosis of solid SPNs with a diameter of 8–30 mm. Some SDCT parameters are also significant in differentiating the pathological subtypes of lung cancer presenting as solid SPNs. This study allowed patients to avoid invasive treatments and examinations. The combination of morphological and quantitative parameters has deepened the understanding of solid SPNs, which can better guide clinicians in developing the most appropriate treatment plan for patients with SPNs.

SPNs can be divided into solid SPN and sub-solid SPN based on the presence or absence of a ground-glass component, which includes pure ground-glass nodules (pGGN) and mixed ground-glass nodules (mGGN) (21). In this study, SDCT quantitative parameters were obtained by delineating the ROI of the solid components of the lesion in the mediastinal window, while there were no solid or less solid components in the mediastinal window in the sub-solid SPN. Therefore, patients with sub-solid SPNs were not included in this study.

Radiological morphology features are important for the diagnosis and the differential diagnosis of SPNs (22, 23). In this study, CT findings of benign and malignant solid SPNs were analysed for 10 aspects, including location, size, lesion shape, lobulation, short spiculation, pleural indentation, vascular convergence sign, air bronchogram, vacuole sign, and calcification. There were significant differences in lesion size, shape, short spiculation sign, and vascular convergence sign between benign and malignant SPNs. Malignant SPNs are larger than benign SPNs because of its malignant characteristics and rapid growth rate. This study shows that lesion size is closely related and an independent risk factor for the prediction of benign and malignant SPNs, which is consistent with previous reports (24, 25). Owing to the heterogeneity of tumour cells, the growth pattern of lesions can be multi-polarised, which also leads to the irregular morphology of malignant tumours (26, 27). The short spiculation sign refers to the radial short thin-line shadow that extends from the edge of the mass to the surrounding lung parenchyma and is not connected with the adjacent pleura (28). Yi et al. reported that the malignant probability of SPNs with a spicular sign is 88%-94% (29). The sign of vascular convergence is also common in malignant SPNs. On the one hand, the cancer focus pulls adjacent blood vessels off the original track and releases a large amount of vascular endothelial growth factor that promotes the formation of new blood vessels, enhances tumour growth, and provides a path for metastasis (30). The lobulation sign, pleural indentation sign, air bronchogram sign, and bubble sign are also important in the diagnosis of malignant SPNs (31, 32). However, this study showed no significant difference between the benign and malignant groups. We hypothesised that since the average size of malignant lesions is 18.225 ± 5.689 mm, which is relatively small; the significance of the imaging parameters could not be achieved. Also, the number of included cases was insufficient.

As a new CT technique, SDCT not only can obtain traditional CT images but also acquire a variety of spectral parameter images (such as VNC, virtual monoenergetic image (VMI), IC, Z_eff_, Uric Acid, and Calcium Suppression). These spectral images can be used to reduce artefacts, improve image quality, reduce contrast agent dosage, and reduce the radiation dose. At the same time, they can also provide more valuable information for the detection of lesions, accurate measurement of lesion size, and differentiation of benign and malignant lesions (33–35).

The application of enhanced CT-derived parameters, CT enhancement value, and enhancement ratio could reduce the background influence caused by machine differences and individual differences and are considered to be effective tools for evaluating tumour angiogenesis. Moreover, CT enhancement values and enhancement ratios are related to microvascular and lymphatic invasion in tumours and can be used as surrogate markers for preoperative detection of lymphovascular invasion (19, 36, 37). Inspired by this, our study will focus, in the future, on the application of SDCT quantitative parameters and their derived parameters after normalisation to evaluate the benign and malignant nature of SPNs more objectively and accurately.

The study showed that between benign and malignant solid SPNs, except for SAR_VNC_ and NED (*P* = 0.114, 0.208), there were significant differences in other SDCT-related parameters (SAR_40keV_, SAR_70keV_, Δ_40keV_, Δ_70keV_, CER_40keV_, CER_70keV_, NEF_40keV_, NEF_70keV_, λ, NIC, NZ_eff_) (*P*< 0.05). The parameters of malignancy were significantly higher which might be because malignant nodules produce a large number of angiogenic factors for tumour growth and stimulate the formation of more microvessels. The increased density of microvessels leads to increased capillary perfusion, and the contrast agent rapidly accumulates after enhancement, such that malignant nodules show a relatively strong contrast enhancement effect. This is consistent with previous related reports (38, 39). Z_eff_ is a quantitative index derived from the atomic number, which represents the composite atoms of compounds or mixtures of various materials and characterizes the tissue composition. The NZ_eff_ value of the malignant SPNs in this study was higher than that of the benign group, which is also consistent with a recent study (40). A possible explanation could be that malignant lesions are densely packed tumour cells with a higher nuclear/cytoplasmic ratio. ROC curve analysis showed that NIC, NEF_70keV_ and NEF_40keV_ were the three best parameters for predicting the diagnosis of benign and malignant SPNs, respectively and the AUC of the NIC were 0.869, 0.854, and 0.853, respectively. This study also showed that NIC still had the best diagnostic performance in differentiating benign from adenocarcinoma SPNs (AUC=0.876) and that NIC also performed well in differentiating benign from squamous cell carcinoma (AUC=0.832). Iodine, as the main component of the contrast agent, directly reflects blood flow and distribution in the intravascular and extracellular spaces. The iodine concentration map is generally considered to have the potential to evaluate the number and blood flow of blood vessels supplying the pulmonary nodules. Using aortic iodine concentration as the standard parameter of NIC minimises the influence of haemodynamic factors on the absolute enhancement of lesions among different individuals, thereby increasing the comparability between different cases and making NIC more specific and stable than other iodine-related indicators. In this study, the NIC cut-off value for differentiating benign and malignant SPNs obtained during the 50 s of enhanced scanning was 0.165, which is in the range of 0.13 and 0.31 under the 25 s and 60 s enhanced scanning time as reported by Wen et al., which was consistent with previous reports (38, 41).

Binary logistic regression analysis, performed on the indicators and parameters that showed statistically significant differences between benign and malignant solid SPNs demonstrated that NIC, Δ_70keV_ and lesion size were independent risk factors for the prediction of malignant SPNs. When the three factors were combined for ROC curve analysis, the AUC for the differential diagnosis of benign and malignant SPNs was 0.903, the cut-off value was 0.728, and the sensitivity, specificity, positive prediction rate, negative prediction rate, and accuracy were 88.2%, 83.3%, 93.8%, 66.7%, and 86.4%, respectively. It was superior to the NIC (AUC=0.869), which had the highest diagnostic efficiency among the independent risk factors. The radiological image features reflect the macroscopic details of the lesion morphology, whereas the quantitative parameters of SDCT reflect microscopic details, such as blood flow and material composition. This study shows that the combination of morphological signs and SDCT quantitative parameters could more accurately reveal the essential characteristics of solid SPNs, thereby further improving diagnostic efficiency, which has rarely been reported in previous studies.

This study has some limitations. First, this was a single-centre retrospective study with a relatively small sample size, and thus larger and multi-centre validation is needed. Second, to reduce the radiation dose, the corresponding SDCT quantitative parameters obtained from venous phase scan images were used in a single phase, although some studies have reported that some parameters (IC, λ, etc.) in the venous phase were better than those in the arterial phase (41, 42), and a multi-phase scanning study to further improve the integrity of the study will be performed, in the future. Third, the radiologists in our centre are experienced in the evaluation of benign and malignant pulmonary nodules, and most of the benign pulmonary nodules are followed up regularly; therefore, the number of benign cases undergoing surgery is small. In the subgroup analysis, the sample sizes of adenocarcinoma and squamous cell carcinoma were small. In the future, a multi-centre collaboration will be considered to increase the sample size.

In conclusion, SDCT quantitative parameters are helpful for the differential diagnosis of benign and malignant solid SPNs, and NIC is superior to the other relevant quantitative parameters. When NIC is combined with lesion size and Δ_70keV_ value for comprehensive diagnosis, the efficacy power could be further improved.

## Data availability statement

The raw data supporting the conclusions of this article will be made available by the authors, without undue reservation.

## Ethics statement

Ethical review and approval was not required for the study on human participants in accordance with the local legislation and institutional requirements. Written informed consent for participation was not required for this study in accordance with the national legislation and the institutional requirements.

## Author contributions

(I) Conception and design: MW, ZZ, WS; (II) Administrative support: MW, ZZ, WS; (III) Provision of study materials or patients: XX, KFL, YX; (IV) Collection and assembly of data: XX, KFL, KL, MW; (V) Data analysis and interpretation: XX, KFL, LZ; (VI) Manuscript writing: All authors; (VII) Final approval of the manuscript: All authors.
